# Race Suicide

**Published:** 1912-03

**Authors:** R. H. L. Bibb


### RACE SUICIDE.

The conservation of our racial integrity, and the study of the forces which operate for or against it, are fast becoming questions of supereminent importance; and as it is only from a moral, a social, a political and an economic standpoint that these forces can be discussed with anything like completeness, the present writing will be limited to a short review of some of the most important of them.

According to the fallacious dogma of Mr. Roosevelt, and those who think as he does upon this subject, the underproduction of human beings as evidenced by the small families of today—about a child and a half, or two, to each family—as compared with the "big," numerous families of a half century ago—families of ten, a dozen, or more—threatens race annihilation more assuredly than any other single cause; and they are "clamoring for a return of the conjugal morality" of former times when the "transient pleasures of men," instead of the "prolonged sufferings to women which the burden of excessive, * * * and often, unwilling * * * child-bearing imposes on them," was the thought of the hour, as a preventive of the threatening race suicide.

There are others who argue that large families—an overproduction of human beings which is, in large measure, responsible for the present high price of living—especially among the poor, who are utterly unable to care for large families—is a much greater menace to racial integrity than is underproduction. They claim that "children who can not be properly kept ought not to be born: * * * that children who are born ought to be properly kept; * * * that the poor should have fewer children—the defective and degenerate * * * none; * * * that no child ought to be brought into the world handicapped by either illness, deformity or want," and that preventive measures—vasectomy, sterilization, for example—looking to these ends, would, from every sociological and political standpoint, be in the interest of racial integrity.

The surplus of such children are thrown on the streets to beg, steal or starve, and go to swell the ranks of pauperism, prostitution and crime, and fill the jails, hospitals and brothels. This is race suicide, and undermines race integrity.

Holding with Prof. Karl Pearson, that you "can not change the leopard's spots; and you can not change bad stock to good; * * * that you may dilute it, possibly spread it over a large area, spoiling good stock, but that until it ceases to multiply it will not cease to be," the more advanced—the more radical, if you like it better—among racial conservators would eliminate the unfit from the birth rate, either by sterilizing, emasculation or by withholding nuptial rites from all those not filling, to completeness, the measure of race perfection, just as breeders (and why not?) of fine stock cull out the unfit from their breeding pens and breed, *only,* from the very best obtainable, in the expectation "that the regulation of births in our families * * * where the progeny from the unfit has been eliminated * * * would give the fewer children born a better chance of survival in greater numbers and in fuller health and efficiency than the children of the old unrestricted families, and of mothers exhausted by child-bearing." They claim that under this system the sad experience with the Juke family in New York never would have disfigured the history of the human family with such an unsightly blotch.

The advocates of the big families of a dozen or more of old times,—which the good Lord always blesses but never feeds, clothes or educates, in their zeal to conserve our racial integrity succeeded in inducing the Federal Legislature to enact a law making it a felony punishable by a fine of $5000, and five years in the penitentiary at hard labor, for any one who imparts any information to any woman whereby she may prevent becoming pregnant. This iniquitous law, which is obeyed best when disregarded most, does not even exempt the poor creature's physician, although she already be the poverty-stricken mother of a half dozen ill-fed, badly clothed, uneducated children, herself also, perhaps, the victim of epilepsy, tuberculosis or some other transmissible disease. In just such cases as this the Neo-Malthusians contend for intervention in the interest of the mother, the children, the father, and in the interest of racial integrity at large.

Injudicious marriages, whereby tendencies to certain diseases, loathsome peccancies, crimes, passions and the neuroses which are transmitted from parent to offspring; improper food, clothing, occupation; the abuse of alcoholics; excessive venery; *coitus interruptus;* abortion; prolonged lactation; intemperate use of tobacco and other narcotic drugs, together with other intemperances, debauches and crimes against nature, of whose baneful influences and fearful ravages the medical profession has been trying, for ages, to warn mankind, are as great, as potent and as pressing today as a menace to race integrity as ever they were; it need not excite surprise, therefore, that many truly conscientious physicians and other philanthropists—sometimes reproachfully designated as "ill-advised propagandists"—should unite in proposing and in urging artificial sterilization—vasotomy or vasectomy, in the male, tubotomy or tubectomy, in the female—*in properly selected cases,* in defense of the integrity of the race.

Those who will go to the trouble to compare the undersized, wizen-faced, hollow-chested, putty-complexioned, wasp-waisted, bottle-rumped and cimblin-headed type so prevalent among the young of today with the type so much in evidence among the young in the days when "every lassie had a laddie" will have no difficulty in seeing how great the change has been; and the question is whether or not artificial sterilization will help in eliminating the one and in perpetuating the other sufficiently to justify its adoption as an aid to race preservation; and if it is, then when, where, how, by whom, and upon whom must it be done?

The future will, doubtless, work out these important problems to the satisfaction and to the best interest of mankind. In the meantime it remains to those of us who are committed to this undertaking to advocate its adoption whenever and wherever opportunity for such advocacy presents itself.

R. H. L. BIBB.

